# A Multichannel Pattern-Recognition-Based Protein Sensor with a Fluorophore-Conjugated Single-Stranded DNA Set

**DOI:** 10.3390/s20185110

**Published:** 2020-09-08

**Authors:** Mari Okada, Hiroka Sugai, Shunsuke Tomita, Ryoji Kurita

**Affiliations:** 1 Health and Medical Research Institute, National Institute of Advanced Industrial Science and Technology (AIST), 1-1-1 Higashi, Tsukuba, Ibaraki 305-8566, Japan; p180218.m-okada@aist.go.jp (M.O.); hiroka.sugai@aist.go.jp (H.S.); 2 Faculty of Pure and Applied Sciences, University of Tsukuba, 1-1-1 Tennodai, Tsukuba, Ibaraki 305-8573, Japan; 3 DAILAB, DBT-AIST International Centre for Translational and Environmental Research (DAICENTER), National Institute of Advanced Industrial Science and Technology (AIST), Central 5-41, 1-1-1 Higashi, Tsukuba, Ibaraki 305-8565, Japan

**Keywords:** biosensors, ssDNAs, multivariate analysis, pattern recognition, proteins

## Abstract

Recently, pattern-recognition-based protein sensing has received considerable attention because it offers unique opportunities that complement more conventional antibody-based detection methods. Here, we report a multichannel pattern-recognition-based sensor using a set of fluorophore-conjugated single-stranded DNAs (ssDNAs), which can detect various proteins. Three different fluorophore-conjugated ssDNAs were placed into a single microplate well together with a target protein, and the generated optical response pattern that corresponds to each environment-sensitive fluorophore was read via multiple detection channels. Multivariate analysis of the resulting optical response patterns allowed an accurate detection of eight different proteases, indicating that fluorescence signal acquisition from a single compartment containing a mixture of ssDNAs is an effective strategy for the characterization of the target proteins. Additionally, the sensor could identify proteins, which are potential targets for disease diagnosis, in a protease and inhibitor mixture of different composition ratios. As our sensor benefits from simple construction and measurement procedures, and uses accessible materials, it offers a rapid and simple platform for the detection of proteins.

## 1. Introduction

The detection and identification of proteins plays an important role in the diagnosis of various diseases [1]. The ‘lock-and-key’ approach, which uses protein-specific molecular probes, such as antibodies [2], aptamers [3], and small organic molecules [4], has been developed to serve this purpose. However, such molecular probes often interact with non-target proteins whose structure is similar to that of the target protein in a cross-reactive manner [5,6,7], which complicates the preparation or synthesis of probes that are completely specific to a target protein. Pattern-recognition-based protein sensing techniques have recently received increasingly attention as alternatives to the lock-and-key approach [8,9,10]; target proteins can be identified using “optical response patterns” generated via non-specific or cross-reactive interactions between proteins and multiple molecular probes. The molecular probes used to construct systems that generate optical response patterns must meet two criteria: (1) Successful interaction via different mechanisms with a variety of proteins, and (2) signal generation, i.e., conversion of the information generated upon interacting into readable optical signals. The requirements for pattern-recognition-based probe design are less stringent than those for specificity-based probes, which is advantageous as it allows the construction of protein-sensing systems via more simple routes. So far, a number of highly sensitive pattern-recognition-based protein sensing systems has been reported that employ materials capable of multi-contact interactions with the macromolecular structure of proteins, such as chromogenic or fluorogenic polymers [11,12,13] and nanoparticles [14,15,16].

In typical pattern-recognition-based sensing systems, individual solutions that contain each molecular probe are placed into multiple compartments (e.g., microplate wells), followed by addition of the protein analyte. Then, the optical response patterns are obtained by recording the optical signal from each compartment. These systems often suffer from the usually time-consuming and error-prone handling of the probes and analytes solutions, as well as from analyte-associated issues due to low sample volumes and adhesion of the biological samples (e.g., cultured cells and biofilms) to the microplate wells. Therefore, in order to broaden the applicability of pattern-recognition-based sensors, sensing systems that address these issues are required. An effective solution is the generation of response patterns from a single compartment; a typical approach involves exploiting the spectral differences, including peak shift and intensity changes, of either single or multiple fluorescent probes. The measurement of these fluorescence changes using multiple detection channels offers an efficient method to obtain the protein optical response patterns. Such “multichannel” sensors have been developed for the pattern-recognition-based detection of proteins and other biological analytes using various materials, including gold nanoparticle–fluorescent protein complexes [17,18], small molecules with multiple fluorophores [19,20,21,22], quantum dots [23,24], and fluorescent polymers [25,26,27]. However, these approaches require the laborious synthesis and preparation of carefully designed organic compounds or recombinant proteins. Therefore, establishing a simple strategy for the construction of multichannel sensors is significant for the practical application of pattern recognition-based sensors.

Single-stranded DNAs (ssDNAs) conjugated with environment-responsive fluorophores are one of the most effective materials for pattern-recognition-based sensors, because sequence and fluorophore customization is flexible and ssDNAs are commercially available at low cost. The segments of ssDNA interact with proteins via various forces (e.g., electrostatic and hydrophobic interactions as well as hydrogen bonding and π-π stacking [28]) in a cross-reactive manner due to the nucleobases and the negatively charged phosphodiester bonds. The fluorophore segments then function by transducing changes in their local environments that result from interactions with the protein. Thus, fluorophore-conjugated ssDNAs are capable of generating differential optical signals following interactions with different proteins. There are a number of sensing systems that employ complexes of ssDNAs and other materials, such as gold nanoparticles [29,30,31], metal oxide nanoparticles [32,33], graphene oxide nanosheets [34,35,36,37,38], and metal oxide nanosheets [38,39]. 

**Figure 1:**
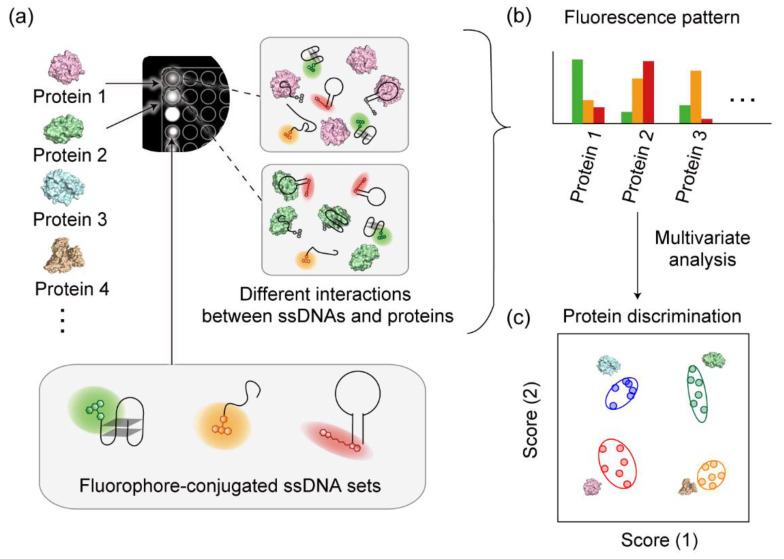
Schematic representation of the multichannel pattern-recognition-based protein sensor with a fluorophore-conjugated ssDNA set. (**a**) ssDNAs conjugated with structurally different fluorophores interact with the protein analytes in a cross-reactive manner within a single well. Measurement of the fluorescence response patterns generated in multiple detection channels (**b**), followed by multivariate analysis to provide accurate discrimination of the protein analytes (**c**).

Recently, we have demonstrated that even in the absence of other complex-forming materials fluorophore-conjugated ssDNAs alone can produce protein-response patterns [40]. As additional materials are not required to form the supramolecular structure, the sensors generate reproducible patterns following a simple preparation route. Herein, we describe a strategy for the incorporation of fluorophore-conjugated ssDNA into a multichannel sensor (Figure 1). A solution containing different ssDNA sequences conjugated with different fluorophores is mixed with the target protein in a single compartment; cross-reactive interactions alter the environment around each fluorophore, which results in a spectral change. The affinity of the protein with the fluorophore-conjugated ssDNAs depends on the DNA sequence and the protein structure, which results in a unique optical response pattern for each protein that is recorded via multichannel detection of the overall fluorescence response. 

## 2. Materials and Methods

### 2.1. Materials

DNA conjugated with carboxyfluorescein (FAM) at the 3′ terminus (DNA-G), carboxytetramethylrhodamine (TAMRA) at the 3′ terminus (DNA-Y), and indodicarbocyanine (Cy5) at the 5′ terminus (DNA-R) were synthesized and purified by Eurofins Genomics (Ebersberg, Germany). Pepsin from porcine stomach mucosa (Pep), thrombin from bovine plasma (ThrB), elastase from porcine pancreas (Ela), α-chymotrypsin from bovine pancreas (Chy), proteinase K from *tritirachium album* (Pro), papain from papaya latex (Pap), trypsin from bovine pancreas (TryB), trypsin from porcine pancreas (TryP), α_1_-antitrypsin from human plasma (AAT), 2-morpholinoethanesulfonic acid (MES), and 3-morpholinopropanesulfonic acid (MOPS) were purchased from Sigma-Aldrich, Co., LLC. (St. Louis, MO, USA). Thrombin from human plasma (ThrH) was purchased from Haematologic Technologies Inc. (Essex, VT, USA). Human serum was purchased from Cosmo Bio (Tokyo, Japan).

### 2.2. General Procedures

The concentration of proteins was determined from the absorbance measured at 280 nm using a Nano Drop 1000 spectrophotometer (Thermo Fisher Scientific, Inc., Waltham, MA, USA) and the extinction coefficients are shown in Table S1. The surface hydrophobicity (*Φ*_surface_) of the proteins was estimated using the sum of the hydrophobicity of the amino acid residues, based on the Miyazawa–Jernigan hydrophobicity scale, divided by the accessible surface area of the protein; calculations were performed using the GETAREA program [41,42]. The solutions were then dispensed into a 96-well half area NBS^TM^ black microplate (Corning Inc., NY, USA) using a PIPETMAX system (Gilson Inc., Middleton, WI, USA). Fluorescence spectra and intensities were recorded using a Cytation5 Imaging Reader (BioTek Instruments, Inc., Winooski, VT, USA).

### 2.3. Fluorescence Spectroscopy Measurements

Solutions (100 μL) containing between 0 and 50 μg/mL protein, 20 nM DNA-G, 20 nM DNA-Y, and 20 nM DNA-R in 20 mM MES buffer (pH = 5.4) were prepared in each well of a 96-well half area plate. After incubation at 30 °C for 10 min, the fluorescence spectra were recorded at 30 °C using three different excitation and emission wavelengths (*λ*_ex_ (nm)/*λ*_em_ (nm): 480/510–540 (DNA-G), 530/575–625 (DNA-Y), and 630/655–700 (DNA-R)).

### 2.4. Pattern-Recognition-Based Sensing and Statistical Analysis 

Solutions (90 μL) containing 22.2 nM DNA-G, 22.2 nM DNA-Y, and 22.2 nM DNA-R in 22.2 mM MES buffer (pH = 5.4) were prepared in each well of a 96-well half area plate. After incubation at 30 °C for 10 min, the fluorescence intensities were recorded at different excitation and emission wavelengths in six different channels (*λ*_ex_ (nm)/*λ*_em_ (nm): 480/520 (Ch1), 515/555 (Ch2), 535/580 (Ch3), 570/610 (Ch4), 630/670 (Ch5), and 665/700 (Ch6)). Next, aliquots (10 μL) of protein in distilled water were added to each well to a final concentration of between 0 and 50 µg/mL protein, 20 nM DNA-G, 20 nM DNA-Y, 20 nM DNA-R, and 20 mM MES buffer (pH = 5.4). After incubation at 30 °C for 10 min, the fluorescence intensities were recorded. The fluorescence responses are presented as *F*/*F*_0_, where *F* and *F*_0_ refer to the fluorescence intensities after and before the addition of protein, respectively. This measurement process was repeated nine times to generate a dataset. The dataset was analyzed using linear discriminant analysis (LDA) and hierarchical clustering analysis (HCA) in SYSTAT 13 (Systat Software Inc., San Jose, CA, USA). 

## 3. Results and Discussion

### 3.1. Design and Characterization of the Fluorophore-Conjugated ssDNA Set 

To develop a pattern-recognition-based protein-sensing system that can accurately identify analytes, it is necessary to design molecular probes capable of producing multivariate differential pattern data that are specific to individual protein analytes. Differences in DNA structure lead to differences in cross-reactivity with proteins [36,40], and are therefore suitable for addressing this issue. Based on the previous report using ssDNAs [40], three sequences with large differences in sequence and conformation, i.e., a simple repeated sequence, a guanine quadruplex-forming sequence, and a hairpin-forming sequence were selected as the structurally diverse ssDNA set (Table 1). Herein, we used a set of molecular probes without inter-probe interactions to simplify the sensor design. In order to generate differential optical patterns from a single compartment, we employed these ssDNAs that were conjugated with three different fluorophores (DNA-G, DNA-Y, and DNA-R). The three fluorophores (FAM, TAMRA, and Cy5) were chosen based on the following characteristics: 1) Their proximity to specific amino acids alters the fluorescence (e.g., tyrosine, tryptophan, histidine, or methionine for FAM and TAMRA [43,44]; tryptophan for Cy5 [45]), and 2) their fluorescence spectra have minimal overlap (*λ*_ex max_ (nm)/*λ*_em max_ (nm): 495/518 (FAM), 555/575 (TAMRA), and 645/660 (Cy5)), which allows selective fluorescence detection. Proteases were chosen as the target protein throughout this study because protease detection is fundamental in the diagnosis and treatment of medical conditions, e.g., cardiovascular and inflammatory diseases as well as cancer [46].

**Figure 2:**
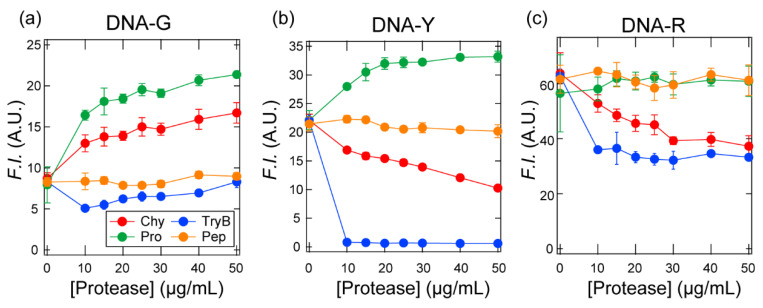
Binding isotherms for 20 nM ssDNAs in the presence of 0–50 μg/mL protease in 20 mM MES (pH = 5.4). (**a**) DNA-G channel, *λ*_ex_ (nm)/*λ*_em_ (nm): 480/519, (**b**) DNA-Y channel 530/580, and (**c**) DNA-R channel 630/668. Values shown are mean values ± standard deviation (*n* = 3).

First, we investigated whether the interactions between the protein analytes and the three fluorophore-conjugated ssDNAs could be converted to individual fluorescence signals. The fluorescence intensity from each fluorophore was recorded after adding different concentrations of the four proteases (Chy, TryB, Pro, and Pep; Table 2) to a solution containing a mixture of the three fluorophore-conjugated ssDNAs. The solution conditions, i.e., 20 mM MES (pH = 5.4), were selected considering that this pH value is effective for transducing the uniqueness of individual proteases into fluorescent signals in fluorophore-conjugated ssDNA systems [40]. The thus obtained titration isotherms showed a marked variation in the fluorescence response from each protease depending on which fluorophore-conjugated ssDNA was detected (Figure 2; for the fluorescence spectra, see Figure S1). For example, the fluorescence intensity corresponding to DNA-G increased with increasing concentrations of Chy and Pro, whereas the DNA-Y fluorescence intensity only increased with increasing concentration of Pro. The fluorescence intensities of all three fluorophore-conjugated ssDNAs decreased in the presence of TryB; in the presence of Pep a noticeable change in fluorescence intensity was not observed. This result likely arises from the electrostatic repulsion between negatively charged Pep and the ssDNAs. Thus, we have successfully converted the unique cross-reactivity of each ssDNA sequence into an individual fluorescence signal. The complex response observed following protease addition may be due to differing affinities with the ssDNAs, or to differences in the local environment surrounding the fluorophore binding site.

### 3.2. Pattern-Recognition-Based Protease Sensing Using a Multichannel ssDNA Sensor 

**Figure 3:**
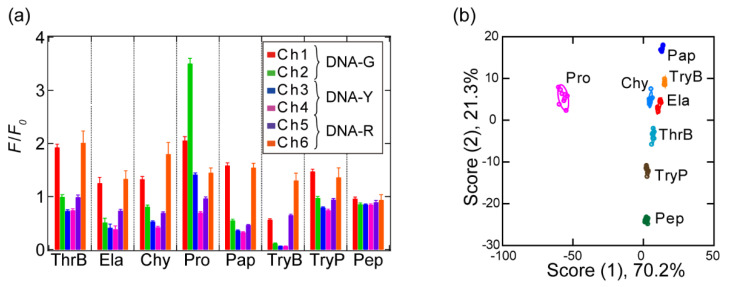
Discrimination of the protease analytes using the multichannel sensor with a fluorophore-conjugated ssDNA set. (**a**) Changes of the fluorescence response upon addition of protease solutions (50 μg/mL) using a sensor containing 20 nM of each of the three fluorophore-conjugated ssDNAs (*λ*_ex_ (nm)/*λ*_em_ (nm): 480/520 (Ch1), 515/555 (Ch2), 535/580 (Ch3), 570/610 (Ch4), 630/670 (Ch5), and 665/700 (Ch6)). (**b**) Discriminant score plot for the protease solutions, where ellipsoids represent the confidence interval (± 1 standard deviation) for each analyte.

After confirming that individual signals from the fluorophore-conjugated ssDNAs could be detected, we subsequently conducted pattern-recognition-based sensing of the protease analytes. For that purpose, we selected eight proteases with a variety of isoelectric points (p*I*) and molecular weights as an initial analyte set (Table 2). In a 96-well plate, a solution containing each protease was mixed with the ssDNAs (20 nM) to a final protease concentration of 50 μg/mL in 20 mM MES (pH = 5.4). The fluorescence responses from each protease/ssDNA combination were recorded using six different channels including shorter and longer wavelengths of *λ*_max_ for each fluorophore in order to obtain a variety of fluorescence responses, and subsequently, a dataset of 6 channels × 8 analytes × 9 replicates was generated. The results are summarized in Figure 3a; as expected, the fluorescence intensities increased or decreased in response to the different protease analytes and detection channels.

To display the complex multidimensional data as two-dimensional graphics, the fluorescence signals were converted into discriminant scores by subjecting the resulting fluorescence signals to LDA [9], which yielded eight discriminant scores, whereby the first two account for the majority of the variation (91.5%) (score (1): 70.2% and score (2): 21.3%), i.e., a plot of these two discriminant scores represents a significant proportion of the fluorescence response. Figure 3b shows the two-dimensional plot of score (1) and (2), where each point represents the optical response pattern from the sensor in response to a single protease analyte. Eight well-separated clusters are observed, corresponding to each protease analyte, indicating that the differences between the fluorescence response pattern of the protease analytes were statistically significant. The seven protease analyte clusters, except the Pro cluster, are close to each other on the score (1) axis, whereas they are isolated on the score (2) and (3) axes (for the plots of score (3), see Figure S2). The dispersion of the clusters over the three discriminant scores suggests the obtained fluorescence patterns contain a large amount of information relating to the properties of the protease analytes.

To understand which factors contribute to the cluster separation for each protease analyte, we examined the correlation between discriminant scores (1) to (3) and the protease characteristics (p*I* and *Φ*_surface_) (Figure S3). Of all the combinations examined, we only observed a relatively high correlation between score (2) and the protein p*I* (*r* = 0.719), suggesting the surface charge of the protease analytes is primarily reflected by score (2). However, on the axis of score (2), the highly cationic TryP cluster (p*I* = 10.2) is close to that of the highly anionic Pep cluster (p*I* = 3.2); accordingly, other forces in addition to electrostatic interactions may contribute to score (2). Score (1) did not correlate with either the p*I* or the *Φ*_surface_. The cluster locations suggest that score (1) reflects a highly specific interaction between Pro and the ssDNAs, in particular DNA-G, for which we observed a significant increase in fluorescence intensity compared to the other ssDNAs. Our sensor can detect a variety of non-specific and highly specific interactions that occur between the ssDNAs and protease analytes, such as electrostatic interactions as well as hydrophobic and hydrogen-bonding interactions, and transform these in fluorescence response patterns.

Next, we evaluated the protease detection reliability of our sensor using two validation tests (a jackknife cross-validation test and a holdout test); here, the entire dataset is divided into “training data” for model creation and “test data” for model evaluation. In the jackknife cross-validation test, one response pattern is removed from the entire dataset (*n* = 9) and treated as test data, while the remaining data are used as training data. This procedure is repeated for all the data. In the holdout test, the training data (*n* = 6) and the test data (*n* = 3) are fixed. In both methods, the test data are individually assigned to the clusters with the closest Mahalanobis distances calculated using the training data. The accuracy is determined based on whether the class of analyte in the test data is assigned to the correct protease in the training data. Both tests provided an accuracy of 100% when all channels were used (Table S2). When less than five channels were used, the accuracy dropped <100% for some combinations; interestingly, we also discovered that even one of the combinations of three channels achieved 100% (Table S3); thus, we have successfully demonstrated that our sensor is capable of the highly reliable detection of eight different protease analytes.

**Figure 4:**
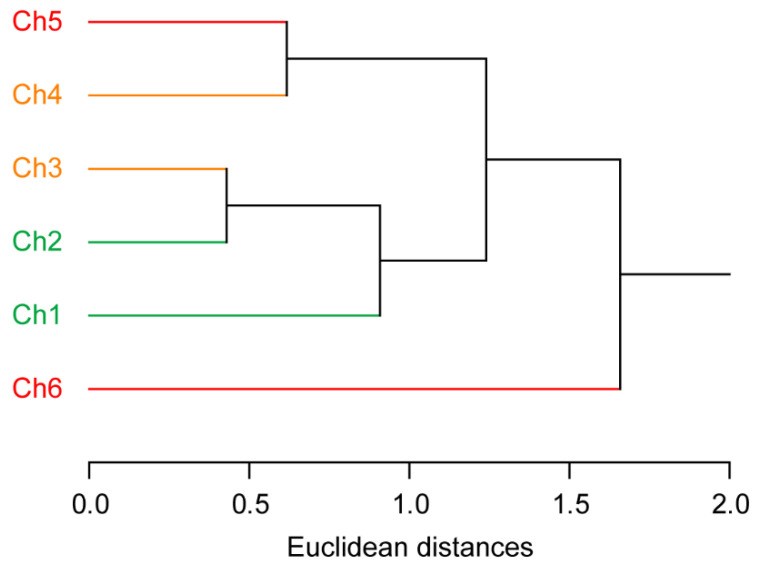
Hierarchical clustering analysis (HCA) of the detection channels of the multichannel sensor with a fluorophore-conjugated ssDNA set. The dendrogram was created based on Euclidean distances using the Ward method and a dataset of 6 channels × 8 analytes × 9 replicates, which was standardized prior to the analysis based on the following equation: *z* = (*x* − *μ*)/*σ*, wherein *z* is the standardized score, *x* is the raw response (*F*/*F_o_*), *μ* is the mean value of the population, and *σ* is the standard deviation of the population. The fluorescence intensities for DNA-G, DNA-Y, and DNA-R were mainly provided using readouts from Ch1 and Ch2, Ch3 and Ch4, and Ch5 and Ch6, respectively.

The detection channels were subjected to an HCA, which suggested that the high reliability of our sensor is due to the effective design of the sensor (Figure 4). This multivariate analysis method determines clusters based on the Euclidean distances between the individual elements of a dataset [9]. The HCA dendrogram shows that the two channels corresponding to the same fluorophore are not clustered (e.g., a combination of Ch1 and Ch2; Figure 4), i.e., the correlation between the responses from the same fluorophore is low. This demonstrates the importance of using different wavelengths to detect each fluorophore signal.

### 3.3. Detection of a Protease and Inhibitor Mixture

To further evaluate the performance of the sensor, a semiquantitative detection was performed on a solution containing a mixture of a protease and inhibitor. We targeted the potential biomarker protein Ela and its inhibitor (AAT) (Table 2). The concentration ratio of Ela to AAT in the blood is usually constant, whereby an imbalance causes various diseases. Clinically, this imbalance is commonly attributed to overexpression of Ela under normal expression of AAT or to underexpression of AAT under normal expression of Ela; the former is observed in the development and progression of cancer [47,48], while the latter is associated with α_1_-antitrypsin deficiency and cystic fibrosis [47,49]. Therefore, protein detection within mixtures containing different concentrations of Ela and a constant concentration of AAT, or vice versa, is critical in the diagnosis of various diseases [47].

**Figure 5:**
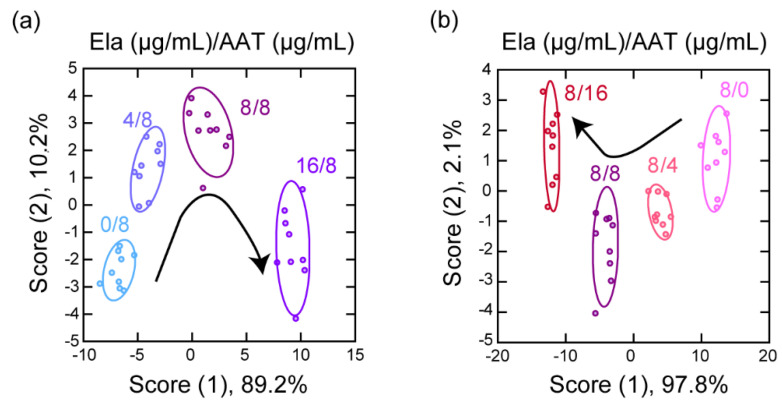
Discrimination of the protein analytes in solutions containing a mixture of Ela and AAT using the multichannel sensor with fluorophore-conjugated ssDNA set. Discriminant score plots for solutions containing (**a**) 0–16 μg/mL AAT with 8 μg/mL Ela and (**b**) 0–16 μg/mL Ela with 8 μg/mL AAT; ellipsoids represent the confidence interval (± 1 standard deviation) for each analyte.

Using our sensor, we acquired a dataset of the fluorescence responses in solution mixtures containing Ela at four different concentrations and a constant concentration of AAT (6 channels × 4 analytes × 9 replicates; Table S4). An LDA of the resulting dataset provided a two-dimensional discriminant score plot, in which the clusters corresponding to the four different ratios of Ela to AAT did not overlap (Figure 5a). In addition, 100% accuracy was achieved for both the jackknife cross-validation and holdout tests (Table S4). Similar results, i.e., cluster separation on the discriminant score plot and high validation accuracy (100% for both tests), were observed when the concentration of AAT was varied and that of Ela was fixed (Figure 5b; Table S5). In both discriminant score plots, the cluster positions shifted monotonically with the score (1) axis with increasing Ela or AAT concentration. High linearity was observed between the changes in score (1) versus concentration ratio (score (1) versus AAT concentration: *r* = −0.981, score (1) versus Ela concentration: *r* = 0.995, Figure S4). When using these as calibration curves, limits of detection (LOD) of 2.7 µg/mL and 2.6 µg/mL were estimated for Ela and AAT, respectively. Therefore, it may be possible to quantitatively analyze Ela and AAT using our multichannel sensor by carrying out a regression analysis based on machine-learning techniques such as support-vector regression [50] and neural-network regression [51].

Using our multichannel sensor, we simultaneously detected the individual fluorescence responses from a mixture of the three ssDNAs, which provided sufficient information to allow discrimination of the protease analytes within a single compartment. In comparison with a non-multichannel sensor, wherein each probe is placed in a single compartment, the multichannel design (*n* probes are placed in a single compartment) allows more rapid measurements (≤ 1/*n* time) using smaller amounts of analyte solution (≤ 1/*n* volume), while maintaining a high discrimination accuracy. In our non-integrated sensor array, the *n* value was 1 [40], whereas that value is 3 in the present case due to the use of three fluorophore-conjugated ssDNAs. Further efficiency improvements can be expected by using more and different fluorophores, whose emission wavelengths do not overlap with those of the fluorophores used in this study. 

A pattern-recognition-based sensing approach has already been applied to the detection of disease-associated compositional changes in clinical samples, such as serum from patients [16,52]. Alternatively, the function of the aptamer sequence can be utilized to selectively detect a specific protease contained in the serum, such as human thrombin (Figure S5). Therefore, we believe that further improvements, including the use of ssDNA sets of aptamer sequences labeled with different fluorophores, will allow our multichannel sensing approach to be applied to complex clinical samples. From a practical point of view, the fluorescence responses obtained by non-specific interactions are influenced by a variety of factors. Therefore, as in many other biochemical assays, the concentration of recognition elements, buffer, temperature, and measurement time should be constant during measurements. The use of regression analysis based on machine-learning techniques enables the quantitative identification of the compositions of analytes based on training data [53] and can therefore be applied to analytes of unknown concentration(s).

## 4. Conclusions 

In summary, we have reported the fabrication of a pattern-recognition-based multichannel sensor using a set of fluorophore-conjugated ssDNAs. This multichannel sensor was used to discriminate between eight different protease analytes and to identify the composition of protease/inhibitor mixtures. We have also demonstrated that fluorophore-conjugated ssDNAs are one of the most advantageous molecular probes for producing optical patterns unique to specific proteins because: (1) A diverse range of moieties allows cross-reactive binding to proteins; (2) flexible design of their sequence and structure is possible, owing to commercial availability; (3) an absence of other materials allows the generation of reproducible optical response patterns. In addition, the multichannel sensor facilitates rapid and simple analyte detection via: Smaller analyte solution volumes, less analyte/probe solution dispensing, and shorter measurement times; the efficiency of this multichannel sensor is three times higher than that of previously reported non-multichannel sensors [40]. Therefore, our pattern-recognition-based multichannel protein sensor not only offers a platform for the high-throughput analysis of proteins or protein mixtures, but also offers scope for application to various analytes, such as those of limited volume or adhesive samples that are difficult to dispense; research focusing on these points is currently in progress in our laboratory.

## Figures and Tables

**Table 1:** The fluorophore-conjugated ssDNA sequences used in this study.

Name	Sequence (5′ to 3′)	Structure
DNA-G	GGTTGGTGTGGTTGG-FAM	G-quadruplex
DNA-Y	TTTTTTTTTTTTTTTTTT-TAMRA	Simple repeated
DNA-R	Cy5-ACGGCATGGTGGGCGTCGT	Stem-loop

**Table 2:** Properties of the proteases and protease inhibitor used in this study.

Analyte	Abbreviation	Source	Molecular Weight (×10^3^)	Isoelectric Point	*Φ*_surface_
Protease					
Pepsin	Pep	Porcine stomach mucosa	35	3.2	0.308
Thrombin	ThrB	Bovine plasma	38	7.1	0.293
Elastase	Ela	Porcine pancreas	26	8.5	0.325
α-Chymotrypsin	Chy	Bovine pancreas	25	8.8	0.285
Proteinase K	Pro	*Tritirachium album*	29	8.9	0.287
Papain	Pap	Papaya latex	23	9.6	0.307
Trypsin	TryB	Bovine pancreas	24	10.1	0.290
Trypsin	TryP	Porcine pancreas	23	10.2	0.312
Protease inhibitor					
α_1_-Antitrypsin	AAT	Human plasma	44	5.4	0.263

## Supplementary Materials

The following are available online at https://www.mdpi.com/1424-8220/20/18/5110/s1, Table S1: List of proteins used in this study; Tables S2, S4, and S5: Dataset of fluorescence response patterns; Table S3: Discrimination accuracies corresponding to each channel combination; Figure S1: Fluorescence spectra of a fluorophore-conjugated ssDNA set; Figure S2: Discriminant score plots for protease discrimination; Figure S3: Correlations between the discriminant scores and the protease characteristics; Figure S4: Linear correlations between the discriminant scores and the protein concentration; Figure S5: Binding isotherms for mixed fluorophore-conjugated ssDNAs upon addition of proteases in the presence of human serum.
