# Supplementary material for: A Multichannel Pattern-Recognition-Based Protein Sensor with a Fluorophore-Conjugated Single-Stranded DNA Set
Source: Sensors (Basel). 2020 Sep 8;20(18):5110. doi: 10.3390/s20185110 (PMC7570997; doi:10.3390/s20185110)
Supplement: Supplementary file 1 [file sensors-20-05110-s001.pdf]

Supporting Information for

A Multichannel Pattern-recognition-based Protein Sensor with a Fluorophore-conjugated Single-stranded DNA Set

Mari Okada [1,2,†], Hiroka Sugai [1,†], Shunsuke Tomita [1,3,*], and Ryoji Kurita [1–3,*]

[1] Health and Medical Research Institute, National Institute of Advanced Industrial Science and Technology (AIST), 1-1-1 Higashi, Tsukuba, Ibaraki 305-8566, Japan.
[2] Faculty of Pure and Applied Sciences, University of Tsukuba, 1-1-1 Tennodai, Tsukuba, Ibaraki 305-8573, Japan.
[3] DAILAB, DBT-AIST International Centre for Translational and Environmental Research (DAICENTER), National Institute of Advanced Industrial Science and Technology (AIST), 1-1-1 Higashi, Tsukuba, Ibaraki 305-8565, Japan.
[†] These authours contributed equally to this work.
[*] Correspondence: s.tomita@aist.go.jp (S.T.); r.kurita@aist.go.jp (R.K.); Tel.: +81-29-861-2634 (S.T.)

Table of Contents

Supporting Tables and Figures

Table S1. Extinction coefficients and PDB ID of the proteases and protease inhibitor used in this study ········ S-2

Table S2. Dataset for the sensing of eight proteases ········ S-2

Table S3. Discrimination accuracies in the sensing of eight proteases ········ S-4

Table S4. Dataset for the sensing of Ela/AAT mixtures with a constant Ela concentration ········ S-5

Table S5. Dataset for the sensing of Ela/AAT mixtures with a constant AAT concentration ········ S-6

Figure S1. Fluorescence spectra of mixtures of fluorophore-conjugated ssDNAs and proteases ········ S-7

Figure S2. Two-dimensional discriminant score plots for the protease analytes ········ S-8

Figure S3. Plots of discriminant scores as a function of p*I* or $\Phi_{surface}$ for the protease analytes ········ S-9

Figure S4. Linear correlations between the discriminant scores and the protein concentration ········ S-9

Figure S5. Fluorescence responses for mixtures of fluorophore-conjugated ssDNAs and proteases ········ S-10

References ········ S-10

Table S1. Extinction coefficients and PDB ID of the proteases and protease inhibitor used in this study.

Analyte	Abbreviation	ε at 280 nm $(mg/mL)^{-1}$ cm^{-1}	PDB ID
Protease			
Pepsin	Pep	1.50 [a]	4PEP
Thrombin	ThrB	1.95 [a]	1UVT
Elastase	Ela	2.02 [a]	1LVY
α-Chymotrypsin	Chy	2.04 [a]	1CHO
Proteinase K	Pro	1.42 [a]	2ID8
Papain	Pap	2.5 [a]	1CVZ
Trypsin	TryB	1.54 [a]	1S0Q
Trypsin	TryP	1.50 [a]	1S81
Protease inhibitor			
α_1-Antitrypsin	AAT	0.45 [b]	1HP7

[a]Taken from the supplier data sheet; [b]Determined based on ref. [1].

Table S2. Data set matrix of the fluorescence intensity ratios (F/F_0) before and after the addition of 50 μg/mL of each protease analyte generated using the multichannel sensor with a fluorophore-conjugated ssDNA set. The far-right column shows the training data (denoted by "-") and the test data as well as the verification result from the holdout test.

Protease analytes	DNA-G		DNA-Y		DNA-R		Holdout test	
	Ch1	Ch2	Ch3	Ch4	Ch5	Ch6	Verification	Accuracy
ThrB	2.001	0.983	0.727	0.727	0.950	1.712	-	-
ThrB	1.884	0.986	0.722	0.757	0.967	1.771	-	-
ThrB	1.992	1.077	0.780	0.792	1.077	2.347	-	-
ThrB	1.860	0.972	0.721	0.733	0.979	1.886	-	-
ThrB	1.907	0.950	0.715	0.705	0.985	1.833	-	-
ThrB	1.899	0.995	0.732	0.724	0.976	2.245	-	-
ThrB	2.013	0.995	0.725	0.753	1.003	2.068	ThrB	Yes
ThrB	1.905	0.971	0.715	0.749	1.016	2.068	ThrB	Yes
ThrB	1.878	1.036	0.726	0.756	0.963	2.182	ThrB	Yes
Ela	1.363	0.596	0.486	0.457	0.754	1.612	-	-
Ela	1.316	0.548	0.446	0.405	0.745	1.145	-	-
Ela	1.305	0.564	0.471	0.421	0.683	1.224	-	-
Ela	1.333	0.563	0.451	0.436	0.763	1.155	-	-
Ela	1.168	0.454	0.350	0.328	0.738	1.400	-	-
Ela	1.142	0.451	0.364	0.338	0.703	1.323	-	-
Ela	1.381	0.609	0.482	0.455	0.733	1.364	Ela	Yes
Ela	1.075	0.403	0.306	0.293	0.760	1.305	Ela	Yes
Ela	1.191	0.430	0.367	0.343	0.741	1.481	Ela	Yes
Chy	1.380	0.792	0.509	0.390	0.651	1.704	-	-
Chy	1.275	0.806	0.535	0.420	0.687	1.576	-	-
Chy	1.311	0.792	0.524	0.403	0.667	1.444	-	-
Chy	1.344	0.841	0.539	0.432	0.717	1.712	-	-
Chy	1.347	0.758	0.511	0.413	0.704	1.922	-	-
Chy	1.269	0.801	0.533	0.418	0.692	1.857	-	-
Chy	1.413	0.827	0.552	0.454	0.703	2.021	Chy	Yes
Chy	1.312	0.815	0.532	0.439	0.698	1.824	Chy	Yes

Chy	1.293	0.852	0.543	0.421	0.693	2.149	Chy	Yes
Pro	2.179	3.662	1.436	0.670	1.013	1.534	-	-
Pro	2.062	3.443	1.384	0.676	0.956	1.288	-	-
Pro	2.175	3.543	1.458	0.690	0.975	1.576	-	-
Pro	1.983	3.613	1.441	0.723	0.978	1.385	-	-
Pro	2.043	3.577	1.405	0.667	0.954	1.529	-	-
Pro	1.996	3.482	1.398	0.708	0.961	1.458	-	-
Pro	2.025	3.379	1.440	0.705	0.987	1.466	Pro	Yes
Pro	2.015	3.469	1.404	0.714	0.924	1.421	Pro	Yes
Pro	2.044	3.403	1.366	0.706	0.937	1.386	Pro	Yes
Pap	1.564	0.541	0.367	0.324	0.457	1.557	-	-
Pap	1.596	0.543	0.362	0.329	0.462	1.508	-	-
Pap	1.598	0.546	0.367	0.327	0.462	1.643	-	-
Pap	1.626	0.582	0.370	0.335	0.484	1.455	-	-
Pap	1.625	0.531	0.358	0.317	0.465	1.644	-	-
Pap	1.584	0.560	0.355	0.324	0.475	1.404	-	-
Pap	1.486	0.532	0.336	0.309	0.476	1.610	Pap	Yes
Pap	1.562	0.589	0.370	0.337	0.450	1.540	Pap	Yes
Pap	1.645	0.549	0.354	0.340	0.450	1.547	Pap	Yes
TryB	0.564	0.120	0.065	0.055	0.622	1.356	-	-
TryB	0.561	0.107	0.065	0.062	0.642	1.226	-	-
TryB	0.550	0.107	0.067	0.069	0.658	1.368	-	-
TryB	0.592	0.132	0.070	0.069	0.664	1.404	-	-
TryB	0.567	0.109	0.067	0.061	0.667	1.226	-	-
TryB	0.555	0.117	0.071	0.063	0.647	1.203	-	-
TryB	0.585	0.127	0.072	0.070	0.676	1.377	TryB	Yes
TryB	0.535	0.118	0.068	0.062	0.625	1.519	TryB	Yes
TryB	0.566	0.119	0.067	0.066	0.656	1.065	TryB	Yes
TryP	1.453	0.965	0.775	0.717	0.955	1.393	-	-
TryP	1.451	0.982	0.781	0.744	0.913	1.215	-	-
TryP	1.433	0.942	0.793	0.746	0.949	1.058	-	-
TryP	1.462	0.940	0.786	0.749	0.927	1.344	-	-
TryP	1.442	0.957	0.780	0.710	0.959	1.333	-	-
TryP	1.469	0.957	0.805	0.741	0.965	1.571	-	-
TryP	1.554	0.974	0.801	0.776	0.963	1.390	TryP	Yes
TryP	1.539	1.022	0.810	0.757	0.943	1.306	TryP	Yes
TryP	1.459	1.010	0.801	0.745	0.905	1.660	TryP	Yes
Pep	0.966	0.856	0.847	0.882	0.856	0.825	-	-
Pep	0.911	0.850	0.841	0.832	0.895	0.787	-	-
Pep	1.012	0.851	0.857	0.843	0.922	0.869	-	-
Pep	0.960	0.853	0.857	0.838	0.855	0.949	-	-
Pep	0.981	0.821	0.840	0.842	0.892	1.018	-	-
Pep	0.952	0.905	0.850	0.848	0.936	0.964	-	-
Pep	0.979	0.853	0.867	0.817	0.900	1.058	Pep	Yes
Pep	0.968	0.831	0.843	0.852	0.942	1.057	Pep	Yes
Pep	0.929	0.882	0.862	0.844	0.869	0.906	Pep	Yes

Table S3. Discrimination accuracies determined by a jackknife cross-validation test in the pattern-recognition-based sensing of eight proteases. The results of the combinations including at least one channel from each fluorophore were examined.

	DNA-G		DNA-Y		DNA-R		Accuracy
	**Ch1**	**Ch2**	**Ch3**	**Ch4**	**Ch5**	**Ch6**	
6 channels	■	■	■	■	■	■	100
5 channels		■	■	■	■	■	100
	■		■	■	■	■	100
	■	■		■	■	■	100
	■	■	■		■	■	100
	■	■	■	■		■	100
	■	■	■	■	■		100
4 channels		■	■		■	■	100
		■	■	■	■		100
	■		■		■	■	100
	■		■	■		■	100
	■	■		■		■	100
	■	■		■	■		100
	■	■	■			■	100
	■	■	■		■		100
		■		■	■	■	99
	■		■	■	■		99
		■	■	■		■	97
	■			■	■	■	97
3 channels		■	■		■		100
	■		■			■	99
	■		■		■		99
		■		■		■	97
		■	■			■	97
		■		■	■		96
	■			■		■	94
	■			■	■		93

Table S4. Data set matrix of the fluorescence intensity ratios (F/F_0) before and after the addition of the Ela/AAT mixture with a constant Ela concentration generated using the multichannel sensor with a fluorophore-conjugated ssDNA set. The far-right column shows the training data (denoted by "-") and the test data as well as the verification results from the holdout test.

Analytes	DNA-G		DNA-Y		DNA-R		Holdout test	
Ela/AAT ratio	Ch1	Ch2	Ch3	Ch4	Ch5	Ch6	Verification	Accuracy
8/0	1.807	1.029	0.936	0.878	0.849	1.117	-	-
8/0	1.679	1.067	0.941	0.919	0.866	1.103	-	-
8/0	1.731	1.008	0.967	0.924	0.841	0.986	-	-
8/0	1.748	1.022	0.907	0.868	0.788	1.128	-	-
8/0	1.696	1.071	0.936	0.914	0.833	1.000	-	-
8/0	1.774	1.103	0.951	0.909	0.821	1.116	-	-
8/0	1.731	1.038	0.912	0.868	0.828	0.940	8/0	Yes
8/0	1.780	1.078	0.936	0.900	0.840	1.103	8/0	Yes
8/0	1.822	1.109	0.970	0.912	0.850	0.985	8/0	Yes
8/4	1.437	1.066	0.903	0.815	0.907	1.157	-	-
8/4	1.419	1.103	0.945	0.895	0.828	1.016	-	-
8/4	1.479	1.065	0.926	0.903	0.849	0.952	-	-
8/4	1.394	1.138	0.918	0.898	0.888	1.053	-	-
8/4	1.436	1.067	0.947	0.900	0.830	1.137	-	-
8/4	1.462	1.105	0.930	0.891	0.853	1.188	-	-
8/4	1.488	1.066	0.916	0.851	0.815	1.176	8/4	Yes
8/4	1.477	1.052	0.906	0.845	0.854	1.209	8/4	Yes
8/4	1.489	1.094	0.953	0.900	0.841	1.071	8/4	Yes
8/8	1.198	1.111	0.890	0.845	0.864	1.236	-	-
8/8	1.146	1.116	0.953	0.862	0.928	1.263	-	-
8/8	1.097	1.095	0.888	0.829	0.835	1.174	-	-
8/8	1.153	1.150	0.929	0.896	0.841	1.378	-	-
8/8	1.192	1.178	0.941	0.915	0.845	1.192	-	-
8/8	1.195	1.064	0.899	0.837	0.853	1.258	-	-
8/8	1.201	1.123	0.909	0.865	0.854	1.235	8/8	Yes
8/8	1.217	1.150	0.910	0.893	0.872	1.191	8/8	Yes
8/8	1.214	1.140	0.929	0.915	0.849	1.313	8/8	Yes
8/16	1.000	1.268	0.961	0.927	0.989	1.559	-	-
8/16	0.952	1.271	0.956	0.905	0.938	1.318	-	-
8/16	0.915	1.233	0.959	0.939	0.899	1.368	-	-
8/16	0.992	1.252	0.965	0.886	0.939	1.426	-	-
8/16	0.966	1.249	0.975	0.954	0.990	1.390	-	-
8/16	1.009	1.249	0.972	0.925	1.025	1.455	-	-
8/16	0.990	1.360	0.987	0.907	0.992	1.614	8/16	Yes
8/16	0.981	1.244	0.961	0.950	0.951	1.553	8/16	Yes
8/16	0.972	1.263	0.973	0.933	0.976	1.565	8/16	Yes

Table S5. Data set matrix of the fluorescence intensity ratios (F/F_0) before and after the addition of the Ela/AAT mixture with a constant AAT concentration generated using the multichannel sensor with a fluorophore-conjugated ssDNA set. The far-right column shows the training data (denoted by "-") and the test data as well as the verification results from the holdout test.

**Analytes**	**DNA-G**		**DNA-Y**		**DNA-R**		**Holdout test**	
**Ela/AAT ratio**	**Ch1**	**Ch2**	**Ch3**	**Ch4**	**Ch5**	**Ch6**	**Verification**	**Accuracy**
0/8	0.894	1.078	0.908	0.836	0.891	0.981	-	-
0/8	0.887	1.077	0.910	0.916	0.859	1.017	-	-
0/8	0.860	1.073	0.901	0.873	0.826	1.014	-	-
0/8	0.911	1.138	0.924	0.883	0.817	0.941	-	-
0/8	0.882	1.172	0.918	0.949	0.868	1.220	-	-
0/8	0.897	1.078	0.921	0.895	0.860	1.000	-	-
0/8	0.923	1.144	0.920	0.908	0.867	0.955	0/8	Yes
0/8	0.912	1.095	0.903	0.936	0.821	0.961	0/8	Yes
0/8	0.857	1.091	0.920	0.906	0.881	0.843	0/8	Yes
4/8	1.080	1.116	0.939	0.909	0.894	1.081	-	-
4/8	1.127	1.152	0.950	0.953	0.880	1.109	-	-
4/8	0.979	1.126	0.933	0.900	0.892	1.105	-	-
4/8	1.013	1.159	0.950	0.915	0.885	1.143	-	-
4/8	1.034	1.216	0.947	0.916	0.882	1.015	-	-
4/8	1.087	1.211	0.942	0.925	0.908	1.216	-	-
4/8	1.073	1.197	0.960	0.907	0.922	1.077	4/8	Yes
4/8	1.009	1.146	0.925	0.912	0.893	1.185	4/8	Yes
4/8	1.074	1.240	0.946	0.974	0.927	1.219	4/8	Yes
8/8	1.198	1.111	0.890	0.845	0.864	1.236	-	-
8/8	1.146	1.116	0.953	0.862	0.928	1.263	-	-
8/8	1.097	1.095	0.888	0.829	0.835	1.174	-	-
8/8	1.153	1.150	0.929	0.896	0.841	1.378	-	-
8/8	1.192	1.178	0.941	0.915	0.845	1.192	-	-
8/8	1.195	1.064	0.899	0.837	0.853	1.258	-	-
8/8	1.201	1.123	0.909	0.865	0.854	1.235	8/8	Yes
8/8	1.217	1.150	0.910	0.893	0.872	1.191	8/8	Yes
8/8	1.214	1.140	0.929	0.915	0.849	1.313	8/8	Yes
16/8	1.190	0.999	0.747	0.700	0.796	1.254	-	-
16/8	1.197	1.002	0.788	0.754	0.817	1.367	-	-
16/8	1.146	0.958	0.783	0.736	0.811	1.222	-	-
16/8	1.218	0.944	0.810	0.748	0.796	1.176	-	-
16/8	1.263	1.031	0.804	0.765	0.792	1.471	-	-
16/8	1.161	0.962	0.772	0.722	0.776	1.293	-	-
16/8	1.201	0.974	0.762	0.724	0.725	1.278	16/8	Yes
16/8	1.127	0.874	0.737	0.689	0.734	1.091	16/8	Yes
16/8	1.229	0.981	0.797	0.756	0.773	1.149	16/8	Yes

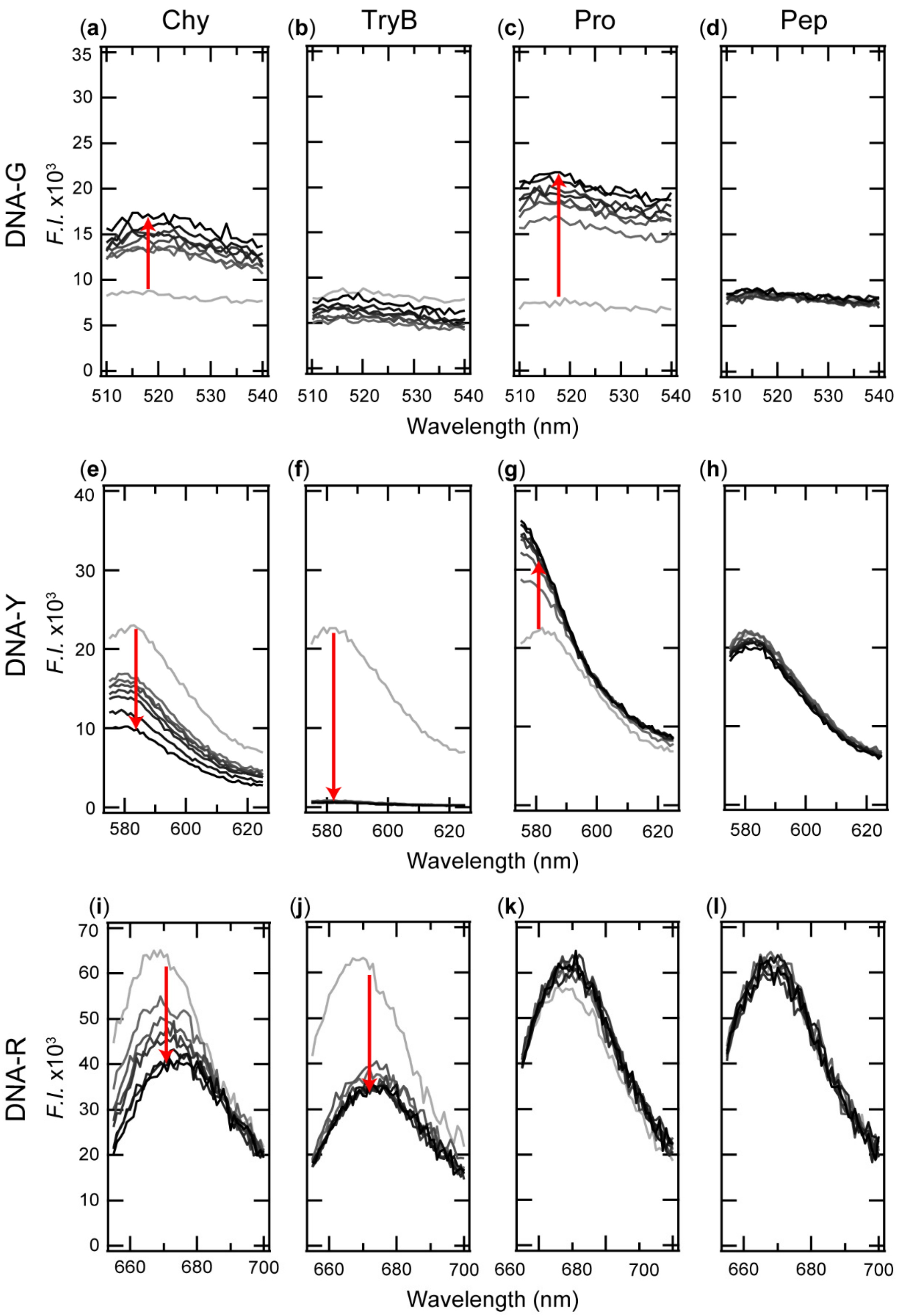

Figure S1. Fluorescence spectra of the mixtures of fluorophore-conjugated ssDNAs and proteases [(**a**, **e**, **i**) Chy, (**b**, **f**, **j**) TryB, (**c**, **g**, **k**) Pro, and (**d**, **h**, **l**) Pep] recorded at the excitation wavelength corresponding to each fluorophore-conjugated ssDNA [(**a**–**d**) DNA-G, (**e**–**h**) DNA-Y, and (**i**–**l**) DNA-R]. Each protease (0-50 μg/mL) was mixed with DNA-G, DNA-Y, or DNA-R, each at a concentration of 20 nM in 20 mM MES (pH = 5.4); λ_{ex} = 480 nm (DNA-G channel), 530 nm (DNA-Y channel), and 630 nm (DNA-R channel).

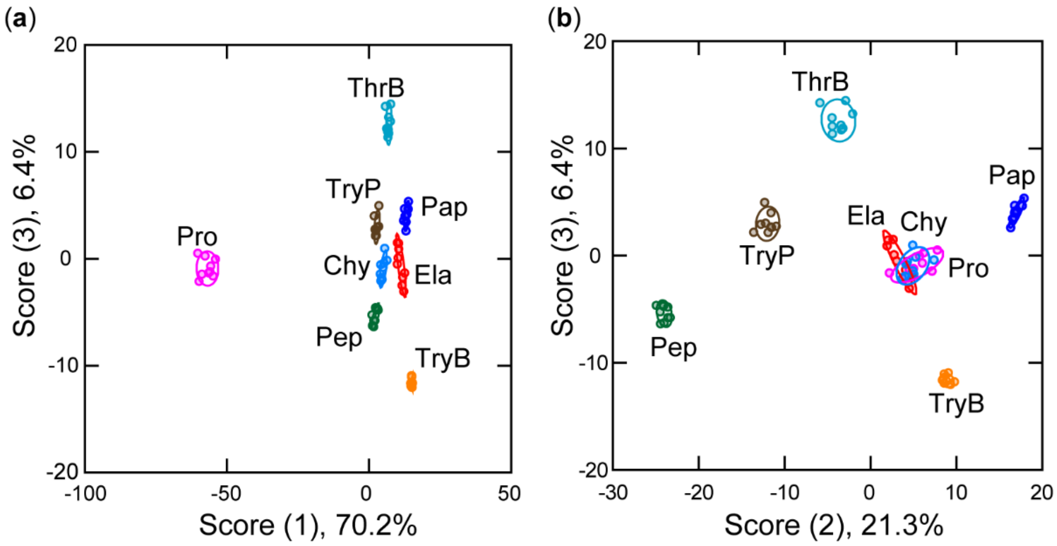

Figure S2. Two-dimensional discriminant score plots for the protease analytes. (**a**) Score (1) versus score (3). (**b**) Score (2) versus score (3). The ellipsoids represent the confidence interval (±1 standard deviation) for each individual analyte.

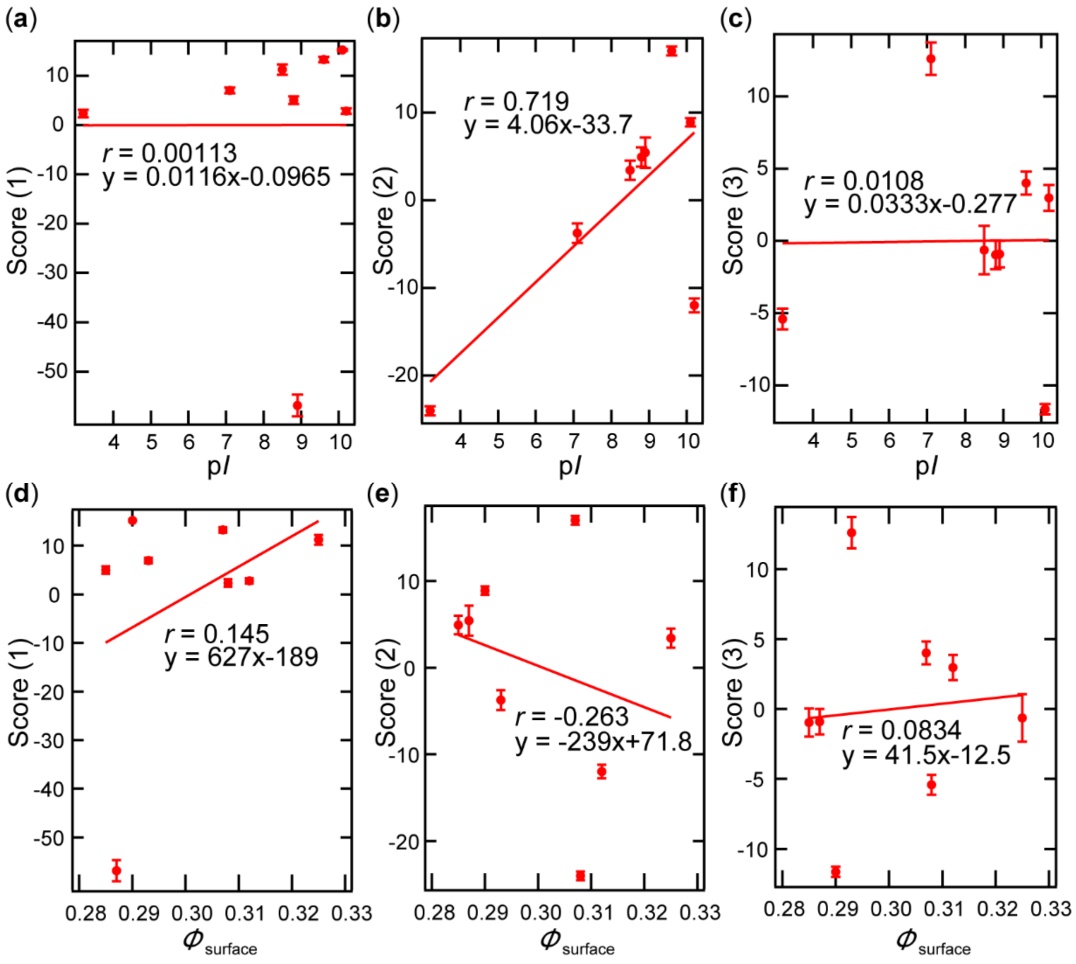

Figure S3. Plots of discriminant scores [(**a, d**) score (1), (**b, e**) score (2), and (**c, f**) score (3)] as a function of (**a**–**c**) p*I* or (**d**–**f**) $\Phi_{surface}$ for each protease analyte. The error bars represent the standard deviation ($n = 9$).

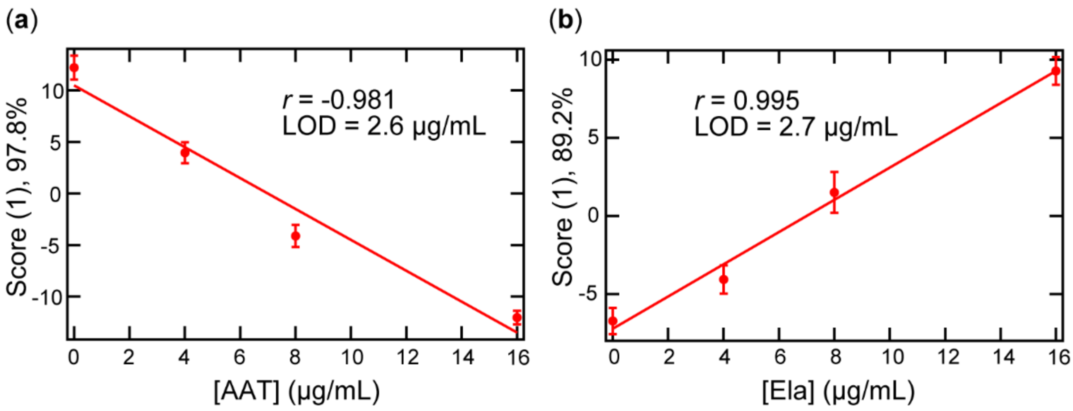

Figure S4. Plots of score (1) as a function of the concentration of (a) AAT and (b) Ela. The error bars represent the standard deviation ($n = 9$). The limit of detection (LOD) for Ela and AAT was determined based on the following equation: LOD = 3.3 (s/S), wherein s is the standard deviation of score (1) at a concentration of 0 μg/mL and S is the slope of the calibration curve.

(**a**)

Analyte	Abbreviation	Source	Molecular Weight(×10^3)	Isoelectric point
Thrombin	ThrH	Human plasma	37	6.4

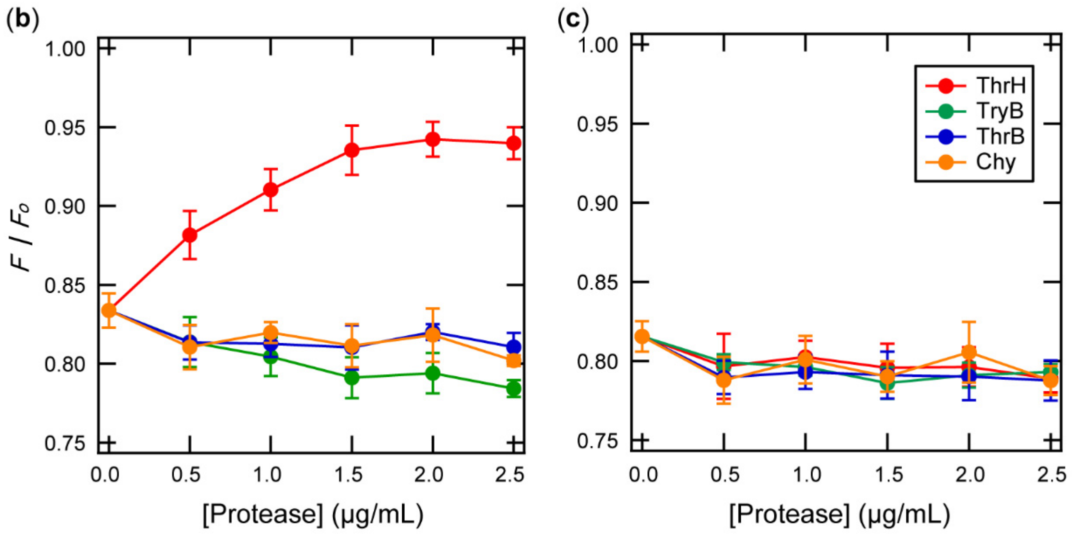

Figure S5. (**a**) Property of thrombin from human plasma (ThrH). (**b-c**) Binding isotherms for the mixture of 20 nM DNA-G (an aptamer sequence that can selectively bind to ThrH) [2] and 20 nM DNA-Y (a simple repeat sequence that exhibits no selectivity to any proteins) in the presence of 0-2.5 μg/mL ThrH with 1% human serum and 20 mM MOPS (pH = 7.4). (**b**) DNA-G channel, λ_{ex} (nm)/λ_{em} (nm): 480/520 and (**c**) DNA-Y channel 535/580. Values shown are mean values ± standard deviation (n = 6). In the presence of 1% serum (containing ~500 μg/mL proteins), DNA-Y exhibited little response to the four protases (Figure S5c), indicating the masking effect due to large amounts of serum proteins. In contrast, a significant fluorescent signal was observed only for ThrH in the case of DNA-G (Figure S5b). These results suggest that the use of aptamer sequences that selectively bind to target proteases can produce information about particular proteases, even from samples in which abundant interferent components are coexisted, as also indicated in our previous study [3]. Given the variety of aptamer sequences known [4], the applicability of our multichannel sensing approach for generating response patterns associated with specific components from complex clinical samples is expected.

References

1. Pace, C.N.; Vajdos, F.; Fee, L.; Grimsley, G.; Gray, T. How to measure and predict the molar absorption coefficient of a protein. *Protein Sci.* **1995**, *4*, 2411–2423.
2. Bock, L.C.; Griffin, L.C.; Latham, J.A.; Vermaas, E.H.; Toole, J.J. Selection of single-stranded DNA molecules that bind and inhibit human thrombin. *Nature* **1992**, *355*, 564–566.
3. Tomita, S.; Sugai, H.; Mimura, M.; Ishihara, S.; Shiraki, K.; Kurita, R. Optical fingerprints of proteases and their inhibited complexes provided by differential cross-reactivity of fluorophore-labeled single-stranded DNA. *ACS Appl. Mater. Interfaces* **2019**, *11*, 47428–47436.
4. Sharma, T.K.; Bruno, J.G.; Dhiman, A. ABCs of DNA aptamer and related assay development. *Biotechnol. Adv.* **2017**, *35*, 275–301.
